# Correlation between the physical activity volume and cognitive and mental capacity among older adult people in China: a cross-sectional study based on the 2020 CHARLS database

**DOI:** 10.3389/fpubh.2024.1462570

**Published:** 2024-11-20

**Authors:** Shixin Li, Jiayi Zhang, Yonghong Yang

**Affiliations:** ^1^Rehabilitation Medicine Center and Institute of Rehabilitation Medicine, West China Hospital, Sichuan University, Chengdu, China; ^2^Key Laboratory of Rehabilitation Medicine in Sichuan Province, West China Hospital, Sichuan University, Chengdu, China

**Keywords:** depressive symptoms, physical activity, older adults, cognitive, risk factor

## Abstract

**Background:**

Currently, due to the progress of the aging population in China and the government’s attention to healthy aging, there is an increasing emphasis on the cognitive function and psychological function of older adult people. Therefore, the aim of our study was to investigate the relationships between physical activity and cognitive and psychological function in order to provide recommendations for exercise guidance.

**Methods:**

The Center for Epidemiologic Studies Depression Scale (CESD-10) was used to assess mental capacity, and cognitive function was evaluated across three domains: orientation, memory and calculation. Total physical activity data were obtained via interviews. Ability of Daily Living (ADL) and Instrumental Ability of Daily Living (IADL) scores were used to determine the presence or absence of daily physical function impairment. Finally, we conducted correlation analysis and logistic regression on participants’ physical activity volume (PAV) and their cognitive and psychological functions, respectively.

**Results:**

A total of 5,871 participants who met the inclusion criteria were selected from the China Health and Retirement Longitudinal Study (CHARLS). The prevalence of depression was 8.1, and 31.6% of the older adult participants reported experienced depressive symptoms. Additionally, 15.6% of the older adult individuals had insufficient weekly physical activity, while 3.9% had daily physical function (DPF) impairment. Physical activity volume (PAV) was negatively correlated with the CESD-10 score, as well as with orientation, calculation, and memory.

**Conclusion:**

Our study confirmed that inactive weekly physical activity was associated with an increased risk of depression, cognitive impairment, and DPF impairment among older adult individuals in China.

## 1 Introduction

The older adult population is a representative component of the age pyramid. As early as 2011, more than 10% of China’s population was over 65. According to the results of the seventh national population census conducted in 2020, the proportion of the older adult population increased by 8.4 percentage points since 2000 when China became an aging society. In the past decade, the proportion of the older adult population has increased by 5.4 percentage points. Among the national population, 264,018,766 people are aged 60 and above, accounting for 18.70%, while 190,635,280 people are aged 65 and above, accounting for 13.50% (1). These figures suggest that the aging population in China has shifted from a short period of relatively slow evolution to an extremely rapid growth stage, and the annual net increase in the older adult population expected to peak in 2023. The issue of the aging population is equally serious worldwide, with the United Nations predicting that adults aged 65 years and above will make up 16% of the world’s total population by 2050 (2). Therefore, all of the above situations highlight the urgency of actively addressing population aging. This growing and dependent population signifies an increased demand for health and social care. A series of health issues, such as depression, cognitive impairment, and cardiovascular disease, tend to increase with age. In turn, this leads to the impairment of activities of daily living and a decrease in quality of life in older adult individuals.

Abnormalities in psychological functioning usually manifest as symptoms of anxiety and depression. Depression and depression symptoms in the older adult population are becoming increasingly common. The prevalence of geriatric depression increased steadily from 1987 to 2012 (3). CLHLS2018 data showed that the detection rate of depressive symptoms in older adult individuals in China was 11.72% (4). However, during the COVID-19 pandemic, the prevalence of depression among older adult individuals in China increased to approximately 26% (5). Previous research has shown that being female, living in rural areas, and having a low level of education level are risk factors for depression, while moderate-to-high physical activity (PA) patterns and social activities are protective factors against depression (6, 7). Psychological symptoms of depression, such as resilience, psychological distress, feelings of hopelessness, personal and interpersonal control and lack of affect, can significantly influence their quality of life (8).

Additionally, in the field of cognitive function, the incidence of mild cognitive impairment (MCI) in older adult Chinese individuals has increased over time. The data from a nationwide survey demonstrated that the prevalence of MCI in older adult individuals in China is 17.1%, and the incidence rate is 70.57/1,000 person-years. The incidence of MCI in China is increasing as the population ages. Overall, if China wants to implement health aging, it can no longer ignore the issues associated with MCI. The early stage of MCI is characterized by dysfunction of multiple cognitive components, the most common of which are immediate memory, associative learning memory, and executive function (9). Older individuals with cognitive impairment are also more prone to concurrent mood disorders, which may be linked to some overlapping biological mechanisms, such as changes in hippocampal atrophy, increased deposition of amyloid *β* plaques, changes in inflammation, and defects in nerve growth factors (10). Fortunately, previous research has provided evidence of improvements in MCI. Lifestyle changes, including alterations in diet/eating patterns, physical exercise, and leisure activities, can reduce the risk of cognitive impairment and dementia. Furthermore, these measures remain effective even in nonagenarians, individuals aged over 90 years (11). According to the WHO, 60% of individual health and quality of life issues are related to lifestyle, which is especially true for older people. Humans cannot stop aging, but they can help them maintain good physical health while aging, and a healthy lifestyle can help them prevent and manage chronic diseases.

The World Health Organization (WHO) put forward the concept of “healthy aging” in the “Report on Global Ageing and Health” in 2015 (12), which aims to promote and achieve the health as well as social participation of older populations. To better achieve healthy aging, the WHO issued the “Integrated Care for Older People guidelines and handbook” (13) for the comprehensive care of older adult people 4 years later, and a number of measures were proposed for the care and management of older adult people, of which physical activity is listed as a strongly recommended measure. The Copenhagen Consensus Statement 2019 (14) suggested that physical activity has a protective effect on physical and cognitive function in older adult individuals. As an important way to promote health management and prevent disease occurrence and dysfunction in older adult people, physical activity alleviates depression through a range of neuromolecular mechanisms. These include enhancing the expression of neurotrophic factors, increasing the utilization of serotonin and norepinephrine, regulating hypothalamic–pituitary–adrenal axis activity, and reducing the systemic inflammatory response (15). For cognitive function, physical activity can lead to changes in the secretion of immunomodulatory cytokines in muscle and adipose tissue, mobilization of regulatory T cells in lymphoid organs, and decrease the number of inflammatory monocytes. These effects collectively reduce the baseline negative effects of inflammation on adult neurogenesis and cognition (16, 17). Previous studies have demonstrated that moderate-to-high-intensity PA patterns are more efficacious than low-intensity PA patterns (18).

This study utilized large sample data from the China Health and Retirement Longitudinal Study (CHARLS) to investigate the associations between varying levels of physical activity and mental and cognitive function in older Chinese adults. The aim of this study was to explore the current status of PAV among older adult individuals in China and to identify the optimal PAV, thereby providing novel data and reference points for mitigating cognitive decline, decreasing the incidence of depression, and enhancing the quality of life for older adult people.

## 2 Materials and methods

### 2.1 Study design

Our study utilized data from the fifth survey (2020) of the China Health Retirement Longitudinal Study (CHARLS) (19). CHARLS is a nationally representative survey that targets Chinese individuals aged 45 years or older and their spouses. It utilizes a multistage sampling method, with probability-proportionate-to-size (PPS) sampling employed at the urban and rural administrative unit sampling stages. The survey encompasses comprehensive assessments of the social, economic, and health statuses of community residents (20). The fifth national survey, conducted starting in 2020 and published in November 2023, encompassed 19,395 respondents. In December 2023, we applied the CHARLS database online, and the study was swiftly approved. In accordance with the objectives of this study, we established the following exclusion criteria for the study subjects: age ≤ 60 years; missing data including: CESD-10 questionnaire, PAV questionnaire, and cognitive-related questionnaire. Based on these exclusion criteria 15,871 subjects were selected from 19,395 patients in the fifth CHARLS follow-up.

### 2.2 Variable selection

#### 2.2.1 Physical activity volume

The CHARLS2020 survey of physical activity volume used a localized abbreviated version of the globally recognized International Physical Activity Questionnaire (IPAQ), a widely used instrument to assess an individual’s level of physical activity. The questionnaire was specifically designed to evaluate the frequency, duration and intensity of physical activity over the past 7 days. Ultimately, we selected the total metabolic equivalent (METs) derived from the IPAQ as a metric to represent the weekly energy expenditure due to physical activity. The calculation of total weekly physical activity using the IPAQ is outlined below. Firstly, the METs values assigned to low-, moderate-, and high-intensity physical activity in the IPAQ are 3.3, 4.0, and 8.0, respectively. The total physical activity (PA) per week was calculated by multiplying the metabolic equivalent (MET) of different PA intensities by the frequency per week (days/week) and the time per day (minutes/day) of each activity. Specifically, data were collected on moderate-intensity physical activities (e.g., weight lifting, tai chi), light physical activities (e.g., walking) and other relevant activities performed by respondents during the previous week. The duration of these activities was categorized as “≤30 min,” “<2 h,” “<4 h,” and “≥4 h.” Since the specific time was not mentioned in the questionnaire, we learned from the processing methods of other scholars and transformed the time range by taking the middle value, namely, “<30 min” was recorded as 30 min, “≥30 min + <2 h” was recorded as 75 min, “≥2 h + <4 h” was recorded as 180 min, and “≥4 h” was recorded as 240 min (20). The PAV score was then calculated by multiplying the frequency of each activity type by the daily physical activity duration index: PAV = 8.0 × total score per week of high activity +4.0 × total score per week of moderate activity +3.3 × total score per week of walking. We defined those with a PAV less than 600 as the inactive PAV group and those with a PAV more than 600 as the active PAV group (21). According to the International Physical Activity Questionnaire (IPAQ), the physical activity level of the older adult individuals was further divided into low-intensity physical activity (<600 METs/min/week), moderate-intensity physical activity (600–3,000 METs/min/week), and high-intensity physical activity (>3,000 METs/min/week). To explore the detailed physical activity volume, we further divided PAVs >600 Met-min/week into six subcategories low-1 (600–1,199), low-2 (1,200–1,799), moderate-1 (1,800–2,999), moderate-2 (3,000–5,999), high-1 (6,000–8,999), and high-2 (≥9,000).

#### 2.2.2 Depression

In the CHARLS 2020 study, the Epidemiologic Studies Depression Scale-10 (CESD-10) was utilized to evaluate common depression symptoms and emotional states based on participants’ feelings over the past week. The total score ranged from 0 to 30. A study conducted in 2014 validated the reliability and validity of the CESD-10 in Chinese community-dwelling older adults (22). Depression was defined by a CESD-10 score > 20. Patients with a CESD-10 score ≥ 20 were assigned to the depression group, those with a score between 10 and 20 (10 ≤ CESD-10 < 20) were assigned to the depressive symptoms group, and those with a score < 10 were assigned to the no depressive symptoms group (23).

#### 2.2.3 Cognitive performance

Considering that the CHARLS2020 did not use the full Mini-Mental State Examination (MMSE), we conducted statistical analysis according to three cognitive domains (orientation, calculation, and memory). In the orientation assessment, participants were required to respond to the current year, season, month, date, and day of the week, and we assign one point to each question. Calculation ability was assessed using the calculation component of the MMSE (24), where participants were instructed to subtract 7 from 100, and then continue subtracting 7 from the result, repeat this process 5 times in total (resulting in the sequence 93, 86, 79, 72, 65). One point was awarded for each correct answer, with a total possible score of 5 points. Memory was assessed by repeating 10 words and recalling them after a few minutes (“river,” “book,” “eye,” “tile house,” “shoulder pole,” “stamp,” “motorcycle,” “grass,” “egg,” “chairperson”). This test uses a variety of delayed recall tests, including the Rey Auditory Verbal Learning Test, WHO-UCLA verbal learning test, California Verbal learning test, etc. Finally, the correct recall of a word was assigned one point, and the final total score was 0–10 points.

#### 2.2.4 Daily physical function

The results of the modified Barthel index and instrumental Ability of Daily Living (ADL) scale of the Lawton questionnaires were partially collected in CHARLS2020. The collected results were divided into four levels, which we assigned as follows: “No difficulty” = 0, “difficult but still able to complete” = 1, “difficult and need help” = 2, and “Unable to complete” = 3. We used some of these items to assess patients’ daily physical function (DPF). The ADL scale extracted the results of dressing, bathing, eating, getting in and out of bed, toileting, and bowel control, and the Instrumental Ability of Daily Living (IADL) scale extracted the results of money management, medication, shopping, cooking, and housework. A final DFP score ≥ 11 was defined as “impaired physical function,” and a score < 11 was defined as “no physical function impairment” (20).

#### 2.2.5 Covariates

**Figure 1:**
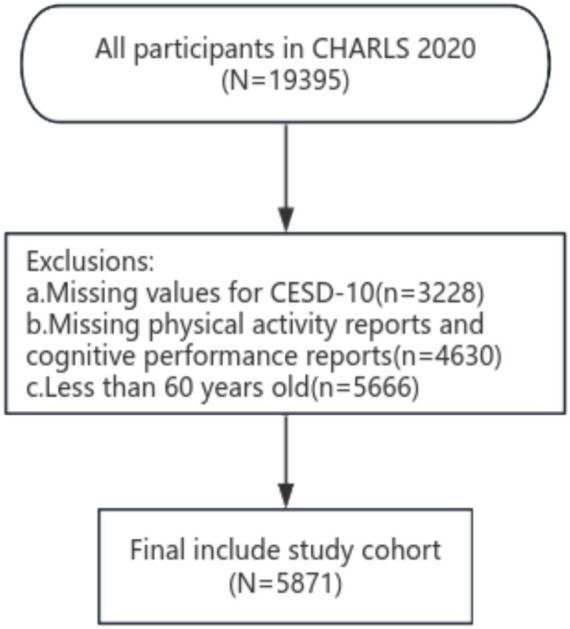
Flowchart of the study participants.

**Figure 2:**
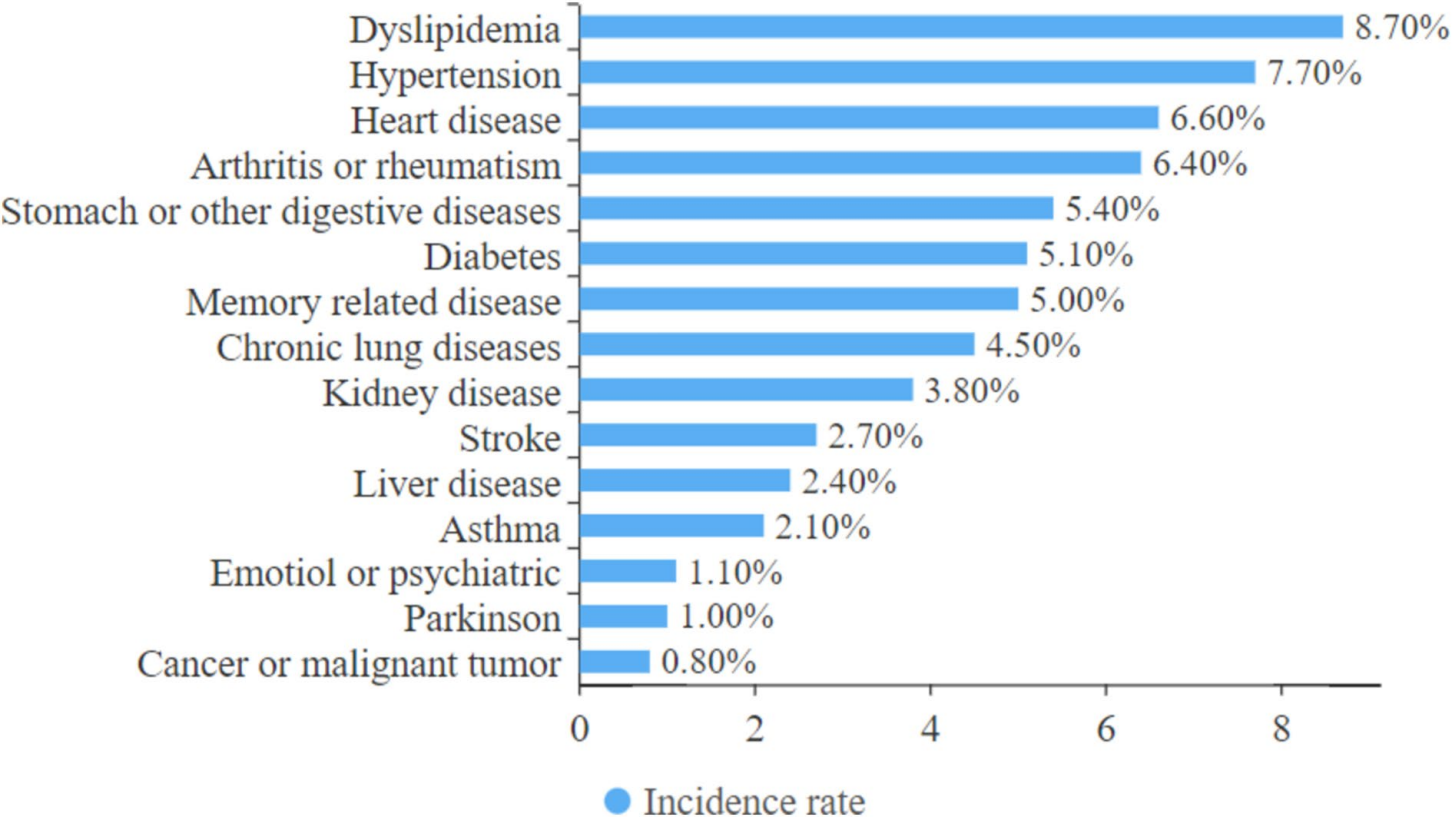
Incidence of common geriatric diseases.

Covariates in this study included (1) demographic variables such as age, sex (male or female), type of residence, and education level (the specific classification is detailed in Figure 1); (2) lifestyle variables, including smoking and drinking habits; (3) chronic disease diagnosis status variables, based on participants’ self-reported history of relevant conditions, including diabetes, hypertension, Parkinson’s disease, cancer, and others. These diseases are characterized by their long duration and typically require ongoing management, as they cannot usually be fully cured.

### 2.3 Data analysis

We used Stata for data cleaning and R version 4.3.2 for data processing. First, we conducted a descriptive statistical analysis on the basic demographic characteristics, physical health and health behaviors of the subjects categorizing them based on their physical activity volume (PAV). Categorical variables are presented in n(%) format, and continuous variables are presented as the mean (M) ± standard deviation (SD). Subsequently, to investigate the effect of weekly PAV on cognitive function, psychological function and activities of daily living in older adult people, we divided all subjects into two groups: those with inactive physical activity (PI group) and those with active physical activity, based on their PAV. A *t* test was used to analyze differences between normally distributed quantitative variables. For comparisons involving non-normally distributed variables, the Wilcoxon test was utilized. Additionally, the χ^2^ test was applied to compare groups and verify the correlation between the included factors and the PAV. The significance level α was set at 0.05. Finally, we used PAV as an independent variable to construct a logistic regression model for multivariate analysis, aiming to determine the impact of PAV on cognitive function, depression. Effect sizes were expressed as odds ratios (ORs) and their corresponding 95% confidence intervals (95% CIs).

## 3 Results

### 3.1 Demographic characteristics

Table 1 displays the baseline data of 5,871 participants, including 2,940 females and 2,931 males. The mean age of the participants was 67.9 years, with a mean CESD-10 score was 9.03. Among the study participants, 73.8% lived in urban areas, 80.1% lived with a partner, 46.6% had a history of smoking, and 34.1% had a history of alcohol consumption (Table 1). Additionally, the CHARLS survey examined the prevalence of 10 common geriatric diseases. Notably, 4,770 participants (81.2%) reported suffering from common chronic diseases (Figure 2). The three most common comorbidities were dyslipidemia (8.8%), hypertension (7.8%), and heart disease (6.6%). Intriguingly, the prevalence of emotional and mental disorders in CHARLS2020 was only 1.1%, which was significantly different from the results of the CESD-10. We hypothesized that this may be due to the stigma of such disorders in traditional Chinese culture and the neglect of mental disorders in the social environment.

**Table 1:** Sociodemographic characteristics of the cohort stratified by physical activity volume.

Variables	Total *N* = 5,871	Inactive PA group	Active PA group	*p* value
Age, *M* ± SD	67.9 ± 5.97	69.4 ± 6.78	65.6 ± 5.76	<0.001
Sex				0.0151
Female	2,940 (50.15%)	500 (54.5%)	2,440 (49.3%)	
Male	2,931 (49.9%)	418 (45.5%)	2,531 (50.7%)	
Residence				<0.001
Rural	4,335 (73.8%)	737 (80.3%)	3,598 (72.6%)	
Urban	1,536 (26.2%)	181 (19.7%)	1,355 (27.4%)	
Marital status				<0.001
Married	4,696 (80%)	672 (81.3%)	4,024 (80.0%)	
Divorced	57 (1.0%)	6 (0.7%)	51 (1.0%)	
Widowed	1,070 (18.2%)	229 (24.9%)	841 (17.0%)	
Separated	24 (0.4%)	6 (0.7%)	18 (0.4%)	
Never married	24 (0.4%)	5 (0.5%)	19 (0.4%)	
Education				<0.001
Elementary school and below	4,023 (68.5%)	710 (77.3%)	3,313 (66.9%)	
Middle school	1,133 (19.3%)	142 (15.5%)	991 (20.0%)	
High school	439 (7.5%)	49 (5.3%)	390 (7.9%)	
Vocational school	168 (2.9%)	12 (1.3%)	156 (3.1%)	
College, college graduates and above	108 (1.8%)	5 (0.6%)	103 (2.1%)	
Poverty				<0.001
Yes	835 (14.2%)	168 (18.3%)	667 (13.5%)	
No	5,036 (85.8%)	750 (81.7%)	4,286 (86.5%)	
Smoking				0.529
Yes	2,733 (46.6%)	443 (48.3%)	2,290 (46.2%)	
No	3,138 (53.4%)	750 (81.7%)	4,286 (86.5%)	
Drinking				<0.001
Yes	2,002 (34.1%)	257 (28.0%)	1,745 (35.2%)	
No	3,869 (65.9%)	661 (72.0%)	3,208 (64.8%)	
CESD-10, *M* ± SD	9.03 ± 6.51	10.46 ± 6.82	8.77 ± 6.41	<0.001
Orientation, *M* ± SD	3.59 ± 1.35	3.19 ± 1.46	3.67 ± 1.32	<0.001
Computation, *M* ± SD	2.93 ± 1.95	2.47 ± 2.02	3.01 ± 1.93	<0.001
Memory, *M* ± SD	4.28 ± 2.64	3.61 ± 2.64	4.41 ± 2.62	<0.001

### 3.2 Factors influencing the level of physical activity among older adults

We grouped the data based on the PAV results for correlation analysis. Our study indicated that several factors were positively associated with increased physical activity levels among the older adult: urban residence, higher education level, being married, younger age, and non-poor household. Among the common diseases in older adult people, Parkinson’s disease is more likely to cause a decrease in physical activity. Notably, the prevalence of inactive physical activity per week among male older adult individuals was 14.2%, which was 2.8% lower than that among female older adult individuals. Older adult residents of urban areas demonstrated a greater propensity for engaging in moderate-to-high physical activity, with an increased probability of 5.3% compared to rural counterparts. Furthermore, our results indicated an intriguing finding: living with up to 15 chronic diseases paradoxically increased the physical activity level of older adult people by 1%. This could potentially be attributed to factors such as the desire to maintain physical function and quality of life despite chronic conditions, although further research is needed to explore this phenomenon in depth.

### 3.3 Association between physical activity volume and depression

Among the older adult participants, 8.1% were diagnosed with depression, while 31.6% experienced depressive symptoms. Women accounted for 60.7% of those with either depression or depressive symptoms. We carried out an analysis to explore the correlations among demographic data, PAV, social activity, smoking and drinking habits, residence status, partner status, three common diseases and other factors in older adult people in both the non-depressed group and the depressed group. Factors with *p* values <0.05 were included in the regression model. As shown in the Table 2 men had a 46% lower risk of depression compared to women. Additionally, engaging in a physical activity volume greater than 600 METs/week reduced the risk of depression more than did the PI group, with the most protective effect observed for activities ranging between 1,200 and 1,799. Furthermore, stroke and Parkinson’s disease significantly increase the risk of depression.

**Table 2:** Factors influencing daily functional problems and depression.

	Depression OR (95%CI)	Daily function problem OR (95%CI)
Physical activity volume		
Inactive PA	Reference	Reference
600–1,199	0.85 (0.67–1.08)	0.42 (0.26–0.68)
1,200–1,799	0.68 (0.56–0.83)	0.46 (0.31–0.68)
1,800–2,999	0.88 (0.71–1.10)	0.09 (0.04–0.21)
3,000–5,999	0.78 (0.65–0.93)	0.16 (0.10–0.26)
6,000–8,999	0.84 (0.68–1.04)	0.09 (0.04–0.21)
≧9,000	0.98 (0.81–1.18)	0.09 (0.05–0.17)
Poverty (No/Yes)		
No	Reference	Reference
Yes	1.87 (1.60–2.18)	1.76 (1.26–2.46)
Partner (No/Yes)		
No	Reference	Reference
Yes	0.69 (0.60–0.79)	1.26 (0.90–1.78)
Residence (Rural/Urban)		
Rural	Reference	Reference
Urban	0.59 (0.52–0.68)	1.37 (0.98–1.93)
Social activity (No/Yes)		
No	Reference	Reference
Yes	0.91 (0.82–1.02)	0.72 (0.53–0.98)

### 3.4 Association between the physical activity volume and daily physical function

**Figure 3:**
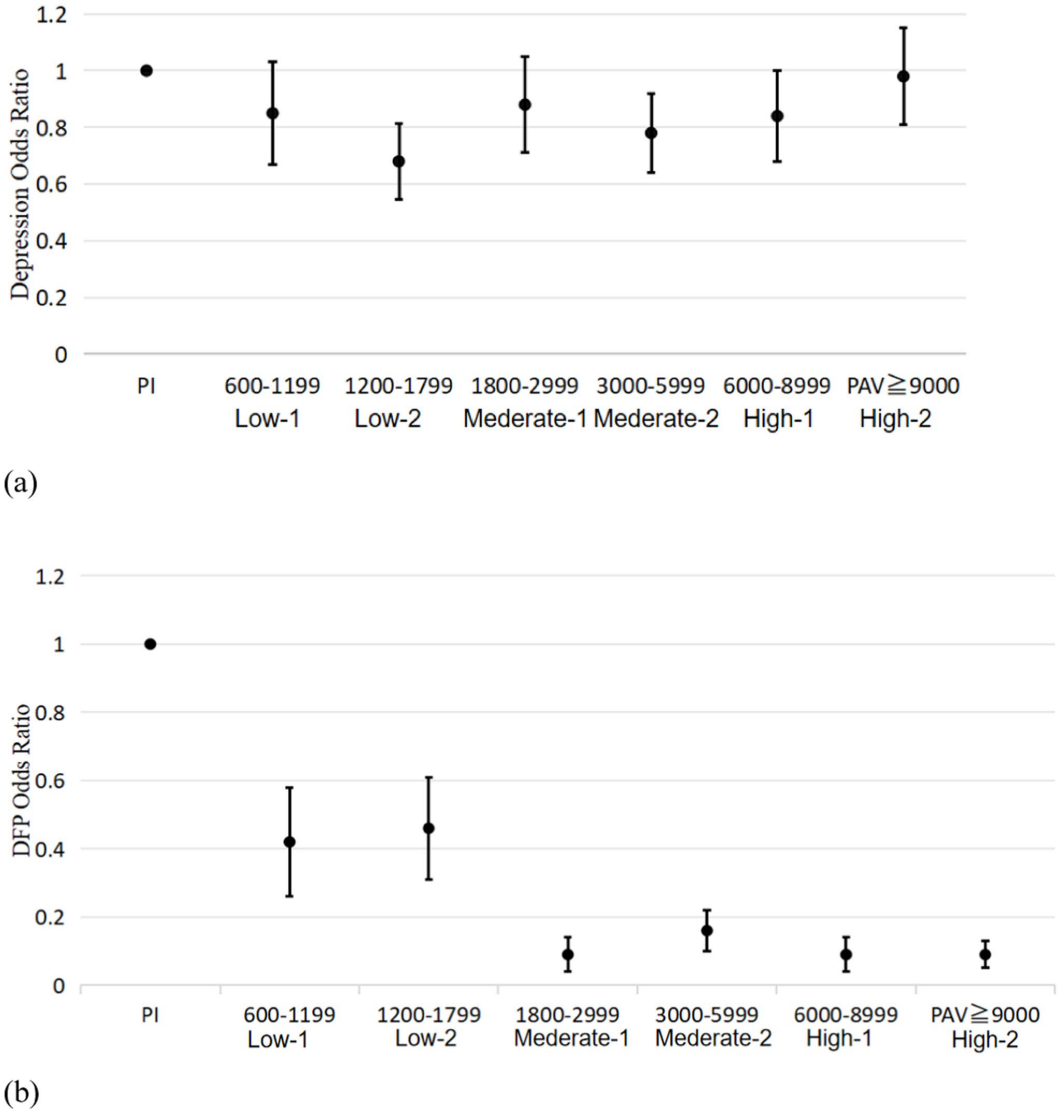
Plot of associations between study participants in different PAV groups with depression (a) or impaired physical function (b).

Among the 5,891 participants we enrolled, daily physical function impairment, was observed in only 231 participants, representing 3.9%. This result indicates that the activities of daily living among Chinese older adult are generally at a good level. According to our statistics, the activities of daily living (ADL) most prone to impairment among Chinese older adult was toileting (16.7%) and for instrumental activities of daily living (IADL), it was household tasks (15.5%). Using the same statistical methods as previously described, we got Binary logistic regression analysis of DPF showed that there was no significant difference in DPF between men and women. Furthermore, The risk of DPF decreased as the PAV increased, with the most beneficial ranges being 1800–2,999 METs/week, 6,000–8,999 METs/week and more than 9,000 METs/week (Figure 3). Participants with depressive symptoms were 4.36 times more likely to have DPF impairment compared to those without depressive symptoms. And the depressed group was 12.32 times more likely to have DPF impairment.

### 3.5 Association between physical activity volume and cognitive performance

**Figure 4:**
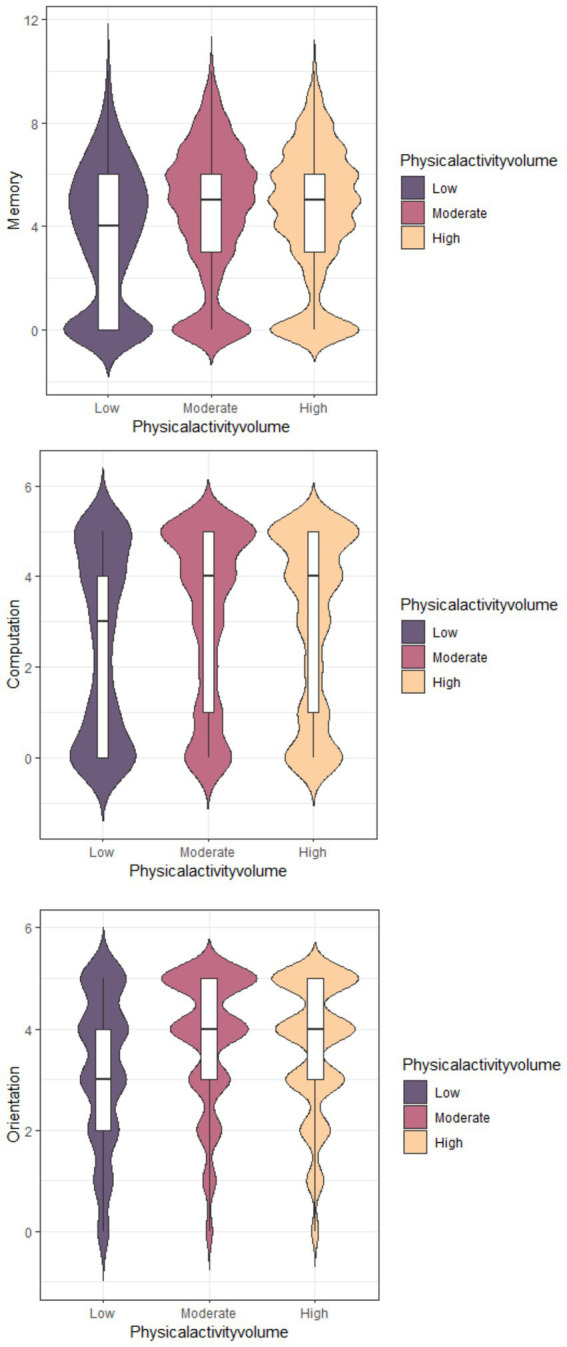
Differences in memory, computation, and orientation among the 3 PAV groups.

**Figure 5:**
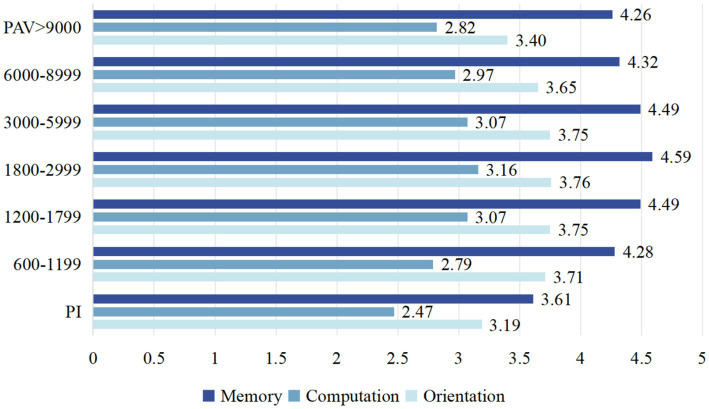
The scores of the seven PAV subgroups in three cognitive domains (memory, orientation, computation).

Among the 5,871 participants we enrolled, 51% older adult population had high total weekly physical activity volume (<3,000 METs/week), 33.2% had moderate physical activity volume (600 < PAV < 3,000 METs/week), and 15.6% had lower total weekly physical activity (PAV < 600 METs/week). Additionally, a violin diagram was created based on the responses to questions about time orientation, computational power, and delayed recall in the CHARLS2020 questionnaire (Figure 4). The low-intensity PAV group had lower memory, computation, and orientation scores compared to the moderate- and high-intensity PAV groups. However, there was no significant difference between the moderate- and high-intensity PAV groups was. To further explore the relationship between physical activity volume and cognitive function, we conducted a subgroup analysis. The bar chart below Figure 5 shows that the group with the best cognitive function had a PAV range of 1,800 to 2,999. Therefore, our study suggests that a lower total amount of activity may be associated with cognitive decline, while a moderate amount of active activity (1,800–2,999 METs/week) may help maintain healthy cognitive function in older adult people.

## 4 Discussion

As demonstrated in our study, the aging population has led to a rising incidence of cognitive impairment in older adult people in China, with a substantial number of individuals already exhibiting cognitive impairment by 2020, resulting in increasing social and health problems. The orienting function is the most vulnerable aspect of cognitive function to impairment. A decline in temporal and spatial orientation will lead to an increase in the risk of being lost. According to the White Paper on the Lost Status of the Older Adult in China, approximately 500,000 older adult people are lost each year, with over 200,000 of them suffering from Alzheimer’s disease. Cognitive decline in older adult people also affect memory, language function, and logical reasoning ability, impairing of daily living activities, and causing a burden on caregivers and society. Mounting evidence highlights the need for solutions to cognitive problems such as orientation, memory, and attention in the older adult population.

Depression, the most common manifestation of mental dysfunction, is also a serious problem in Chinese older adult people. Compared with previous studies, which used CESD-10 > 10 (25) as the standard of symptoms of moderate-to-severe depression (MSD), referring to Lina Zhou2021’s study, we set the criteria for depression at CESD-10 > 20 (26), which is more suitable for Chinese older adult individuals. According to these criteria, 8.1% of the 5,871 older adult individuals in our study had depression, and 31.6% had depressive symptoms. Although we only sampled a portion of the Chinese older adult population, the above data also suggest that the depression problem in the Chinese older adult population may be more serious, and routine depression screening in the older adult population may need to be strengthened in subsequent studies in China. Our study found a positive correlation between depression and cognitive function, with depressed participants having lower cognitive function scores. This finding is consistent with several other studies that have demonstrated an association between depression and cognitive decline in the older adult population. Depression and cognitive impairment often coexist, and both subthreshold depression (with depressive symptoms) and full-fledged depression can lead to cognitive impairment. Among all cognitive components, attention and working memory are the most seriously affected. Studies have used the GDS to evaluate the severity of depressive symptoms in older adult people and have shown that there is a significant negative correlation between GDS scores and attention and working memory (27).

In our study, we found that both the depressive symptom group and the depressed group had worse scores on cognitive components, including temporal orientation, delayed recall, and numeracy. Participants in the depressed group had even worse scores on cognitive function. These data support the hypothesis that the more severe depressive symptoms are in the older adult population, the worse their cognitive function will be. Depression and cognition can independently and separately influence human health, but they also interact and are interdependent. Complex interrelationships between them determine health, activities of daily living and quality of life. We found that participants with depression or depressive symptoms had a 4.36 times higher probability of developing activities of daily living dysfunction compared to normal participants. Additionally, the cognitive function score of participants with activities of daily living dysfunction was significantly lower than that of normal participants. Therefore, the occurrence of depression and cognitive dysfunction significantly affects the quality of life of older adult people and increases the burden on caregivers and society.

Therefore, we pondered whether a method that can simultaneously prevent and improve depression and cognitive dysfunction might enhance the daily living abilities and quality of life of older adult pondered in our country. Previous studies have demonstrated that engaging in appropriate physical activity can effectively stave off declines in intrinsic capacity among the older adult, encompassing cognitive function, motor function and mental function (28). From a physiological perspective, physical activity has a number of benefits, including the prevention of decreased cardiovascular system function, sarcopenia, reduced bone density, and hypertension, among others. The underlying mechanism may lie in the fact that exercise elevates the level of anabolic hormones in older adult people (29). Previous research has demonstrated that physical inactivity is an independent risk factor for various diseases, including cardiovascular disease and respiratory disease. A global survey assessing the insufficiency of physical activity and the prevalence of noncommunicable diseases estimated that insufficient physical activity accounts for 6% of coronary heart disease cases, 7% of type 2 diabetes mellitus cases, 10% of breast cancer cases, and 10% of colon cancer cases worldwide (30). An epidemiological survey revealed that the total risk ratio associated with exercise was 1.46 (95% CI, 1.22–1.75) for people with PI and 1.16 (95% CI, 0.84–1.59) (31) for people with high PAV. Moderate to high PAV was inversely associated with CVD mortality, with the best results associated with ≥35.5 MET/h of physical activity per week (≈60–75 min of moderate-intensity physical activity per day) (32). Similarly, using CHARLS2020 data, we found that the total weekly activity of Chinese older adult people was significantly negatively correlated with the occurrence of depression, positively associated with cognitive function and AD. Inactive PA is associated with an increased risk of age-related diseases (such as stroke, Parkinson’s disease, hypertension, among others), as well as cognitive function decline, anxiety and depression. PAV > 600 METs/week significantly reduces the risk of physical, mental, and cognitive dysfunction in older adults. Among them, a PAV within the range of 1,800–2,999 MET-minutes/week has been found to more effectively reduce the risks of physical and cognitive impairments and depression in Chinese middle-aged and older adult people. Additionally, we observed that PAV tends to be higher in older adults residing in urban areas in our study. We are inclined to attribute this to the more widespread, in-depth, and popular adoption of health education in urban settings, which has subsequently heightened the awareness of the importance of adequate physical activity among older adult urban residents.

A study conducted among older adults in Singapore has revealed that disability, depression, loneliness, and the frequency of contact with friends were significant mediators of the relationship between cognitive scores and well-being (33). A decrease in social activities and feelings of loneliness are also crucial factors impacting the quality of life of older adult individuals. Although our study did not uncover a significant correlation between social activities and the functional status of older adult people, physical activity can serve as a valuable form of social engagement for them. Additionally, physical exercise fosters communication and interaction among older adult peers, thereby alleviating loneliness and social isolation, and enhancing peer support. In the future, we may consider designing group therapy that integrate physical activity and social interaction specifically tailored for the older adult population to improve their function.

Therefore, we recommend promoting an active lifestyle. A 2021 retrospective study, which compared six types of exercise—aerobic exercise, resistance training, mind–body exercise (such as yoga), racquet sports (such as tennis and basketball), combat sports, and dance—found that, for the purpose of improving cognitive function, resistance training was the most effective in enhancing certain cognitive dimensions (34). The Finnish Geriatric Intervention Study to Prevent Cognitive Impairment and Disability (FINGER) trial involved a two-year intervention that included nutritional guidance, exercise, and other strategies, and found it to be effective in slowing cognitive decline (35). A randomized controlled trial currently being conducted by Andrew Pipingas and colleagues in Australia investigates whether an intervention combining walking and the Mediterranean diet can reduce cognitive decline in older adults. This provides a new perspective for enhancing the physical activity volume (PAV) in older adult individuals (36). For older adult people in China, walking is the most accessible physical activity, requiring no special threshold. Perhaps we can also explore the optimal walking intensity and duration that is most suitable for older adult people in China. In terms of exercise function, aerobic training and resistance training have been found to increase the abundance of NAMPT protein in human skeletal muscle, thereby helping to prevent a decline in exercise function in older adult individuals (37). Similarly, traditional Chinese exercises such as tai chi and Baduanjin have also demonstrated comparable benefits. However, for older adult people who have contraindications to certain types of exercise, the choice of exercise must be carefully considered. In such cases, it is advisable for rehabilitation professionals to formulate personalized exercise prescriptions.

## 5 Conclusion

In summary, our study focused on exploring the relationships between physical activity volume (PAV) and depression, as well as cognitive function in the older adult Chinese population. We found that physical activity was negatively correlated with the severity of depressive symptoms. On this basis, we further investigated the optimal range of PAV for the older adult individuals in China and identified some suitable exercise training methods tailored for this population. Therefore, we recommend that future should delve deeper into personalized physical activity/exercise training programs that are most suitable for older adult people in China. Specifically, the frequency, intensity, duration, type, total amount and progression of physical activity/exercise training should be regulated through exercise prescriptions, guiding older adult people to engage in physical activity more reasonably and mitigating potential risks associated with exercise training. This approach could also indirectly alleviate the long-term burden and costs of healthcare in aging societies. However, it should be noted that our study was a cross-sectional study, which could not establish a causal relationship between physical activity and cognition or depression. Additionally, CHARLS2020 did not include a comprehensive Mini-Mental State Examination (MMSE) survey, preventing a definitive diagnosis of cognitive impairment. Therefore, we hope that future studies will continue to follow up their follow-up on the data, with a particular focus in depressive symptoms, cognitive function, and physical activity, to provide stronger evidence of a causal relationship between physical activity and these factors.

## Data Availability

Publicly available datasets were analyzed in this study. This data can be found here: https://charls.pku.edu.cn.
